# Radiologic overview of sinonasal lesions

**DOI:** 10.3389/fradi.2024.1445701

**Published:** 2024-08-30

**Authors:** Mohammed U. Syed, Steve J. Stephen, Akm A. Rahman

**Affiliations:** ^1^ University of Rochester School of Medicine and Dentistry, Rochester, NY, United States; ^2^ Department of Imaging Sciences, University of Rochester Medical Center, Rochester, NY, United States

**Keywords:** sinonasal malignancy, adenocarcinoma, esthesioneuroblastoma, rhabdomyosarcoma, sinonasal undifferentiated carcinoma, squamous cell carcinoma, sinonasal melanoma, sinonasal lymphoma

## Abstract

Sinonasal tumors are often malignant and comprise approximately 3% of all head and neck malignancies. Half of these tumors arise in the nasal cavity, and other common locations of origin include the ethmoid and maxillary sinuses. Some unique clinical features are anosmia and altered phonation but the most common general features include headache, epistaxis, and diplopia. CT and MRI may be used to assess tumor location, invasion of adjacent tissue, presence of metastasis, internal tumor heterogeneity, and contrast enhancement. Local invasion of the tumor beyond the sinonasal tract can impact adjacent structures such as the cranial nerves, skull base, branches of the internal carotid artery, and orbit leading to neurologic signs, facial pain, and diplopia. Imaging is used in the diagnosis, staging, and treatment planning of sinonasal tumors. This collection of benign and malignant sinonasal tumors will include some rare and unique cases with an emphasis on imaging features demonstrating a wide variety of pathologies.

## Introduction

Relative to benign lesions, malignant sinonasal tumors are more common, more likely to present later in the disease course, and more often associated with poor prognosis (1). Sinonasal cancer comprises 3% of all head and neck malignancies with an incidence rate of 0.56 per 100,000 and a male:female ratio of 1.8:1. The most common anatomical sites for sinonasal lesions include the nasal cavity (43.9%), the maxillary sinus (35.9%), and other paranasal sinuses (2).

**Figure 1:**
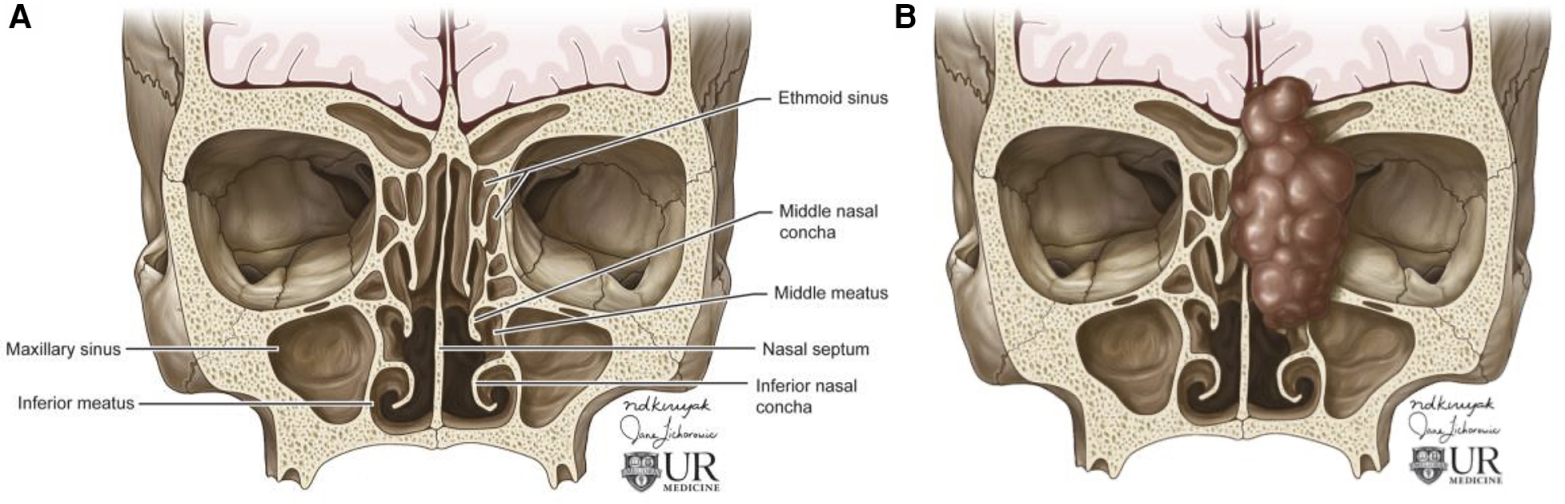
Overview of sinonasal anatomy. Coronal illustrations of normal sinonasal tract anatomy **(A)** and abnormal paranasal sinuses depicting an invasive sinonasal tumor extending from the nasal tract into the skull base, concha, orbit, and maxillary sinus **(B)**. Figure 1 was made for this article by University of Rochester employed illustrators. Permission was given by the illustrator for use in this article.

The sinonasal tract consists of the nasal cavity and paranasal sinuses. The nasal cavity contains a midline nasal septum and 3–4 bilateral bony conchae with underlying meatuses to drain the paranasal sinuses. The paranasal sinuses extending from the nasal cavity are lined with respiratory epithelium and consist of four bilaterally paired sinuses: maxillary, sphenoid, frontal, and ethmoid. The frontal, maxillary, anterior ethmoid, and middle ethmoid sinuses bilaterally drain to the middle meatuses below the middle conchae (Figure 1). The posterior ethmoid sinuses bilaterally drain to the superior meatuses below the superior concha. The sphenoid sinuses bilaterally drain to the spheno-ethmoid recesses above the superior conchae. The converging paranasal sinus drainage pathways into the nasal tract provide an avenue for the local spread of sinonasal tumors. Additionally, the sphenoid sinus lies inferomedial to the internal carotid artery, optic nerve, pituitary gland, and cavernous sinus while the maxillary sinus is just anterior to the pterygopalatine ganglion as well as the maxillary artery, vein, and nerve. These structures in close proximity to the paranasal sinuses may be adversely affected by the growth of sinonasal tumors.

Histologically, most malignant sinonasal tumors are classified as squamous cell carcinoma (51.6%). Other classification groups include adenocarcinoma (12.6%), sarcoma, melanoma, lymphoma, and sinonasal undifferentiated carcinoma (SNUC) (1, 2). Imaging is an important aspect of the diagnosis, staging, and longitudinal assessment of both benign and malignant sinonasal tumors (1). The sinonasal tumors presented in this article begin with malignant epithelial tumors including squamous cell carcinoma, adenocarcinoma, adenoid cystic carcinoma, sinonasal undifferentiated carcinoma, and esthesioneuroblastoma. These lesions are followed by other malignant tumors including neuroectodermal (melanoma), lymphoma, soft tissue (rhabdomyosarcoma). Then, more commonly benign lesions are presented including soft tissue (meningioma), epithelial (inverted papilloma), antrochoanal polyp, mucous retention cyst, salivary gland type adenoma (pleomorphic adenoma), and germ cell (dermoid cyst). This article aims to present at least one tumor from each histological tumor type based on the classification presented by Bacigliano and colleagues (3).

Many sinonasal tumor types exhibit differentiating imaging features on CT and MRI such as anatomical location, invasion of adjacent tissue, metastasis, tumor homogeneity, internal signal intensity, and contrast enhancement. Imaging is essential to evaluate the tumor grade, extent of invasion, perineural spread, and presence of necrosis (1). CT is more effective for imaging local invasion into bone and identifying intralesional calcifications as well as feeding arteries. Characteristic patterns of bone invasion include extensive bone destruction for high grade malignancies and slow, expansile bone growth for benign lesions or low-grade malignancies (1). MRI is more effective for greater contrast resolution, differentiating soft tissue regions of a tumor, and detecting perineural spread as well as dural invasion. Malignant tumors are non-specifically hyperintense on T2 weighted imaging and iso-hypo intense on T1 weighted imaging. These imaging characteristics can be used to determine pre-treatment prognosis, select treatment modalities, and evaluate tumor stage (1).

Additionally, apparent diffusion coefficient (ADC) mapping from diffusion weighted MR imaging (DWI) can assist in the diagnosis of sinonasal tumors, particular types of malignancy, and the tumor stage. Higher ADC values indicate fluid, hypocellularity, cartilage, or mucus which may be more common with benign sinonasal lesions, while lower ADC values are indicative of hemorrhage, hypercellularity, or abscess, which may be implicated in malignant sinonasal tumors (1, 4). While ADC may be a potential differentiator between benign and malignant lesions, this review will focus on the anatomic impact of sinonasal tumors and affected regions.

The purpose of this article is to provide an exploration of a variety of malignant and benign sinonasal tumors with a focus on imaging characteristics and anatomic impacts to offer a concise reference for radiologists in training. To this end, the key information about each tumor type presented including incidence, anatomic locations impacted, and general imaging findings is briefly summarized in Table 1.

**Table 1:** Summary of presented sinonasal lesion characteristics.

Sinonasal lesion type	Incidence & epidemiology	Common anatomic locations	General imaging findings
Squamous Cell Carcinoma	51.6% of all sinonasal malignancies (2).	Maxillary sinus, nasal cavity, and ethmoid sinus with local invasion and aggressive bone destruction (1).	Larger tumors exhibit internal signal heterogeneity. CT for assessing extent of local invasion and bone destruction. T1: Isointense, moderately enhanced on T1 + C. T2: Hyperintense (1).
Adenocarcinoma	Consists of many histological subtypes. Accounts for 10%–20% of sinonasal tumors (1, 5).	Intestinal-type (ITAC) can arise from ethmoid sinus, nasal cavity, and maxillary antrum with local invasion to adjacent structures (5).	Occasional areas of calcification reflecting mucin content which alters MRI signal intensity. T1: Gradual contrast enhancement with higher mucin content. T2: Hyperintensity with higher mucin content. Iso-hypointense with lower mucin-content (1).
Adenoid Cystic Carcinoma	Adenocarcinoma subtype. 10% of salivary gland cancers, 5% of sinonasal malignancies (6).	Maxillary sinus and nasal cavity. Possible local extension. Lymph node spread and metastasis to lung, liver, and bone (1, 5).	Irregular, heterogeneous mass on MRI T1: Isointense T2: Iso-hyperintense depending on the amount of cellularity (1, 6).
Sinonasal Undifferentiated Carcinoma (SNUC)	3%–5% of sinonasal cancers (7).	Superior nasal cavity and ethmoid sinus. Location invasion and distant metastases possible (1, 7, 8).	Ill-defined, non-calcified mass. Central necrosis on CT with contrast. T1: Isointense with heterogeneous contrast enhancement T2: Iso-hyperintense (1, 8).
Esthesio-neuroblastoma	0.4 per million incidence, 2%–6% of intranasal tumors (9).	Arises from the superior nasal cavity. Local invasion to paranasal sinuses, anterior cranial fossa, cribriform plate and orbit. Metastasis to cervical lymph nodes, lungs, liver, and bone (9).	Possible incidental intracranial lesions. CT for analyzing local invasion and bone involvement. MRI for identifying subtle extent of local invasion, dural seeding, subarachnoid seeding, and metastases. T1: Hypointense T2: Hyperintense (9).
Melanoma	0.5%–2% of malignant melanomas and 4% of all head and neck melanomas (10, 11).	Nasal cavity, nasal septum, inferior and middle turbinates, lateral nasal wall, and maxillary sinus. Regional and distant metastasis are common (11).	Strong, heterogeneous contrast enhancement on MRI with contrast. T1: Melanin or hemorrhage appear iso-hypointense. Amelanotic appears hypointense. T2: Melanin or hemorrhage may appear iso-hypointense. Amelanotic appears hyperintense (11, 12).
Lymphoma	**NHL:** 2nd most frequent sinonasal malignancy. Often in adults >60 and 7%–19% of cases show regional lymphadenopathy (1, 13).	**NKTL**: Nasal cavity **DLBCL**: Maxillary sinus with intracranial or orbital involvement (14).	**NKTL**: Heterogeneous, intratumoral necrosis, marked contrast enhancement **DLBCL**: Homogenous, mild contrast enhancement (14). **NHL**: Intratumoral sinus wall remnants on CT if permeative bone invasion across sinus wall is present. Lower ADC in maxillary sinus differentiates from SCC. T1WI: Isointense T2WI: Hyperintense and homogeneous contrast enhancement (1).
Rhabdomyosarcoma	3%–5% of all childhood tumors (15)	Parameningeal (50%), non-parameningeal (25%), and orbital (25%). Parameningeal may arise near skull base structures (15).	CT with contrast: Isodense to hypodense and homogeneously contrast enhancing. T1W1: Isointense to muscle T2W2: Moderately hyperintense to muscle “Botryoid Enhancement Pattern” seen on MRI (16) DWI shows lower mean ADC compared to SCC (17).
Meningioma	24%–30% of intracranial tumors. Ectopic including sinonasal meningioma account for <1% of non-epithelial tumors (18).	May arise from ectopic foci disconnected from cranial nerve foramen, vertebral canal, or intracranial tissue (18).	CT: Uniform density, clear boundary, polyp-like structure, homogenous contrast enhancement (18).
Inverted Papilloma	0.4%–4% of sinonasal tumors (19).	Locally aggressive, originating from the lateral nasal wall near the middle turbinate or ethmoid recesses, but can extend into the maxillary sinus, ethmoid sinus, and nasopharynx (19).	CT for soft tissue mass and bone remodeling “Convoluted cerebriform” pattern on MRI as alternating high and low signals on T2 and T1 weighted MRI with contrast (19, 20).
Antro-choanal Polyp	Most often seen in children with allergies, but also in adults with chronic sinusitis (21).	Arise from the maxillary sinus and pass through the ostia to the choana and nasopharynx (21).	CT: Unilateral hypodense mass from an enlarged opacified maxillary sinus. T1: Iso-hyperintense T2: Iso-hypointense & peripheral enhancement with contrast administration (21).
Mucous Retention Cyst	1.4% to 9.6% prevalence. Most common incidental finding in the maxillary sinus (22).	Paranasal sinuses without extension into nasal cavity (22).	T1: Variable to hypointense T2: Hyperintense (22).
Pleomorphic Adenoma	0.4% incidence rate. Most common in females ages 30–60 (23).	Nasal septum (23).	T1: Hyper-intense CT: Calcifications.
Dermoid Cyst	1 in 20,000 to 1 in 40,000 individuals. (24) 11%–12% of head and neck dermoid lesions. (25).	Midline of nasal bridge from glabella to columella with potential intracranial extension. (24, 25).	Tracts through sinonasal bone may be present. T1: Hyperintense CT: Low-density.

## Sinonasal squamous cell carcinoma

**Figure 2:**
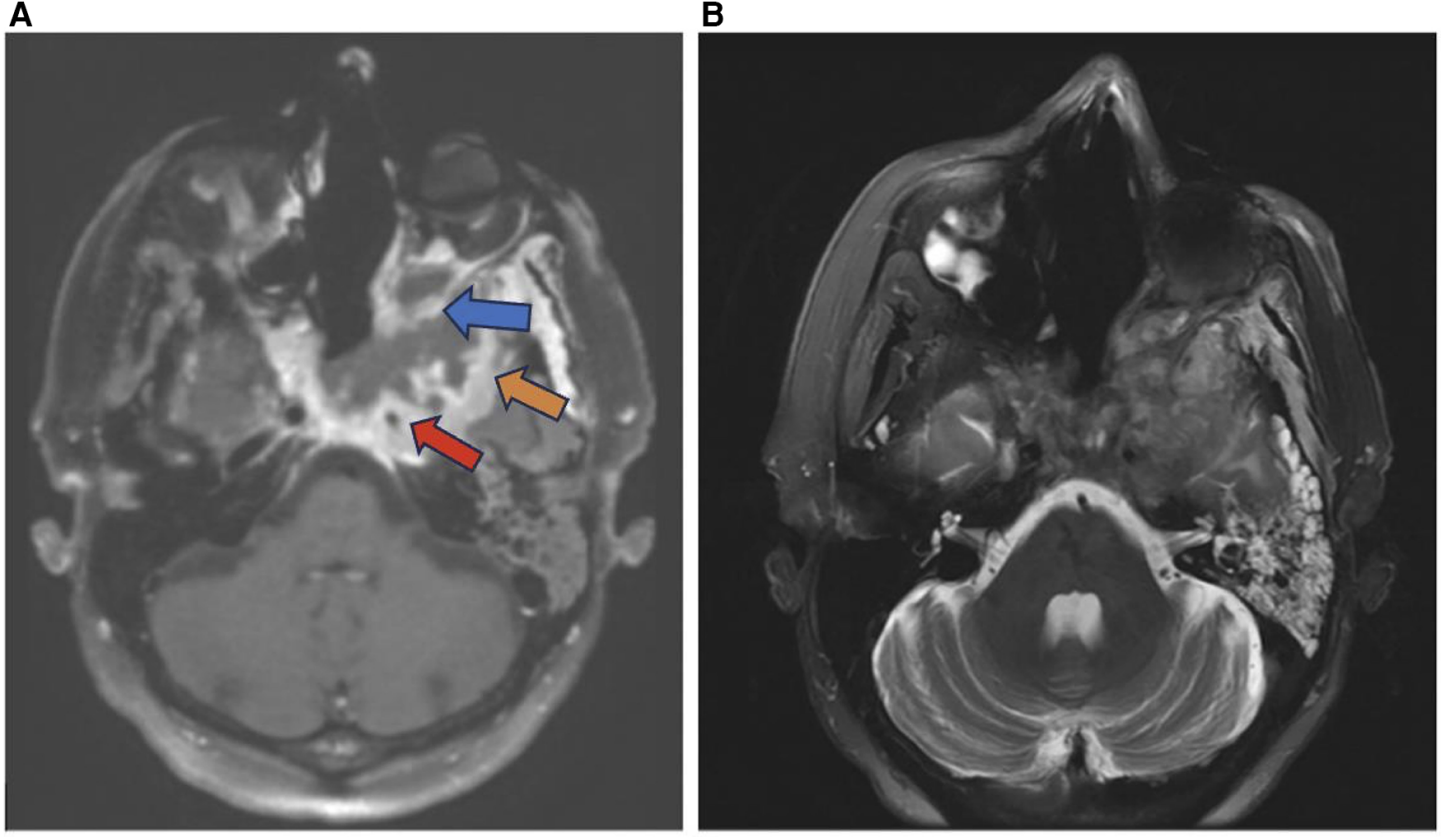
Squamous cell carcinoma. Axial T1 + C **(A)** and axial T2 **(B)** weighted images demonstrate invasive sinonasal squamous cell carcinoma centered in the sphenoid sinuses (red arrow) extending to the adjacent orbit (blue arrow), left middle cranial fossa (orange arrow), and clival region.

Sinonasal tumors are more often malignant than benign (1). Sinonasal squamous cell carcinoma (SCC) is the most common subtype of sinonasal carcinoma accounting for 51.6% of malignant sinonasal tumors (2). The incidence of sinonasal SCC has been declining from 1973 to 2009, but the overall survival rates have not improved with 20 year overall survival of 30.68% for men and 26.35% for women (26). SCC most frequently affects the maxillary sinus, nasal cavity, and ethmoid sinus and is characterized by aggressive bone destruction of the adjacent sinus walls as well as local invasion into the opposite sinonasal area, orbital wall, infratemporal fossa, and skull base at more advanced stages (Figure 2). Larger SCC tumors are associated with hypoxic stress leading to internal signal heterogeneity and characteristic regions of intratumoral necrosis. SCC tumors are non-specifically isointense on T1 weighted MRI, faintly hyperintense on T2 weighted MRI, and moderately enhanced on contrast-enhanced T1 weighted MRI. Contrast-enhanced CT may be helpful for evaluating the extent of bone invasion resulting from local tumor extension (1).

Well known risk factors for squamous cell carcinoma include tobacco smoking and exposure to occupational carcinogens such as softwood and leather dust. Additionally, viral oncogenesis may play a role in sinonasal SCC development and progression (27). Chowdhury et al. found 16 of 26 (62%) sinonasal tumor biopsy samples were positive for human papilloma virus (HPV) (28). In another retrospective analysis, Doescher et al. found that only Epstein-Barr virus (EBV) positive sinonasal SCC patients (20/44) developed lymph node or distant metastases further suggesting a role for viral oncogenesis in the progression of sinonasal SCC (29).

## Adenocarcinoma

**Figure 3:**
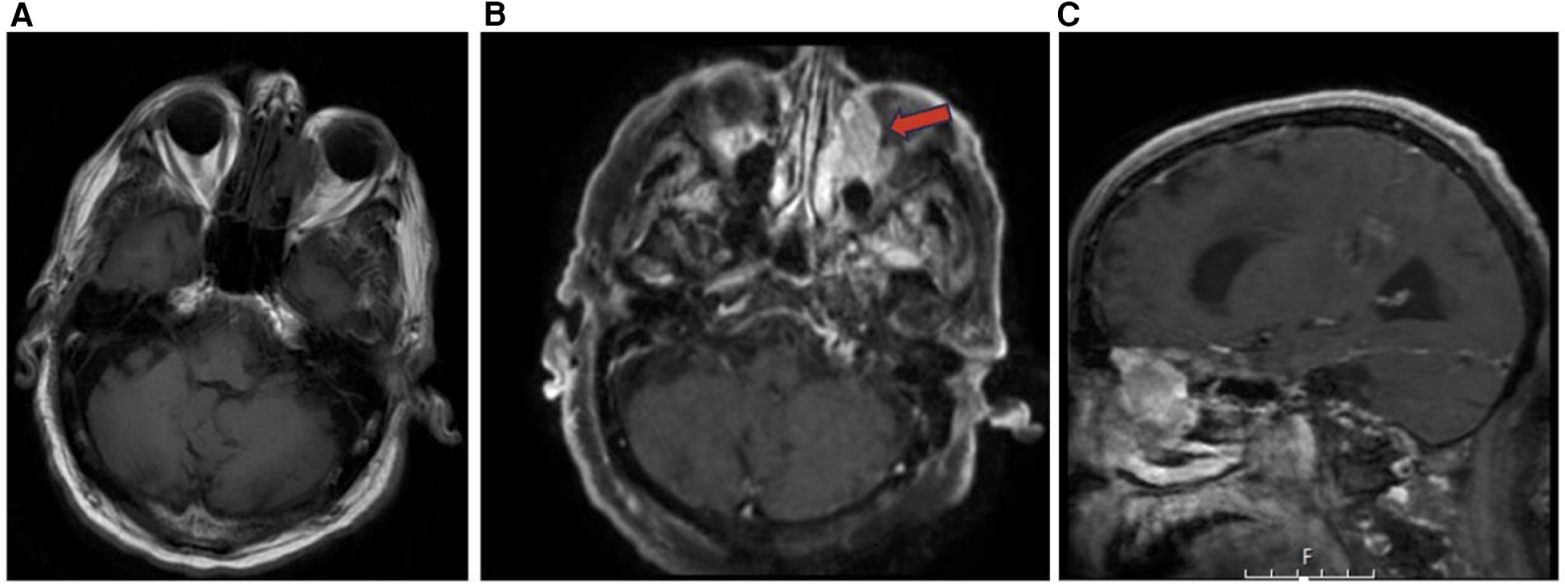
Adenocarcinoma MRI axial T1 **(A)** axial T1 + C **(B)** and sagittal T1 + C **(C)** of an invasive sinonasal carcinoma in the left ethmoid to extraconal orbital region (red arrow) likely representing a primary sinonasal adenocarcinoma.

Many histological subtypes of sinonasal carcinoma including adenocarcinoma display locally invasive characteristics involving structures adjacent to the sinonasal tract including the brain, orbit, cranial fossa, nasopharynx, and internal carotid arteries (Figure 3). This can cause diplopia, facial pain, or neurologic signs. Invasive sinonasal adenocarcinoma accounts for 10%–20% of sinonasal malignancies (1). Sinonasal intestinal-type adenocarcinoma (ITAC) exhibit locally invasive characteristics and involve the ethmoid sinus (40%), nasal cavity (25%), and maxillary antrum (20%) (5). Colonic-type is the most common ITAC and exhibits wide extension into the orbit, pterygopalatine fossa and cranial cavity. Sinonasal ITACs most commonly affect males averaging 50–64 years old and have 1,000 times greater prevalence in workers exposed to hardwood dust. Wood-dust related ITACs more commonly arise in the ethmoid sinus while sporadic ITACs more commonly arise in the maxillary antrum (5). Higher mucin content adenocarcinomas exhibit hyperintensity on T2WI as well as contrast enhancement on T1WI with contrast. Sinonasal ITAC may have a similar imaging presentation to squamous cell carcinoma (1).

## Adenoid cystic carcinoma

**Figure 4:**
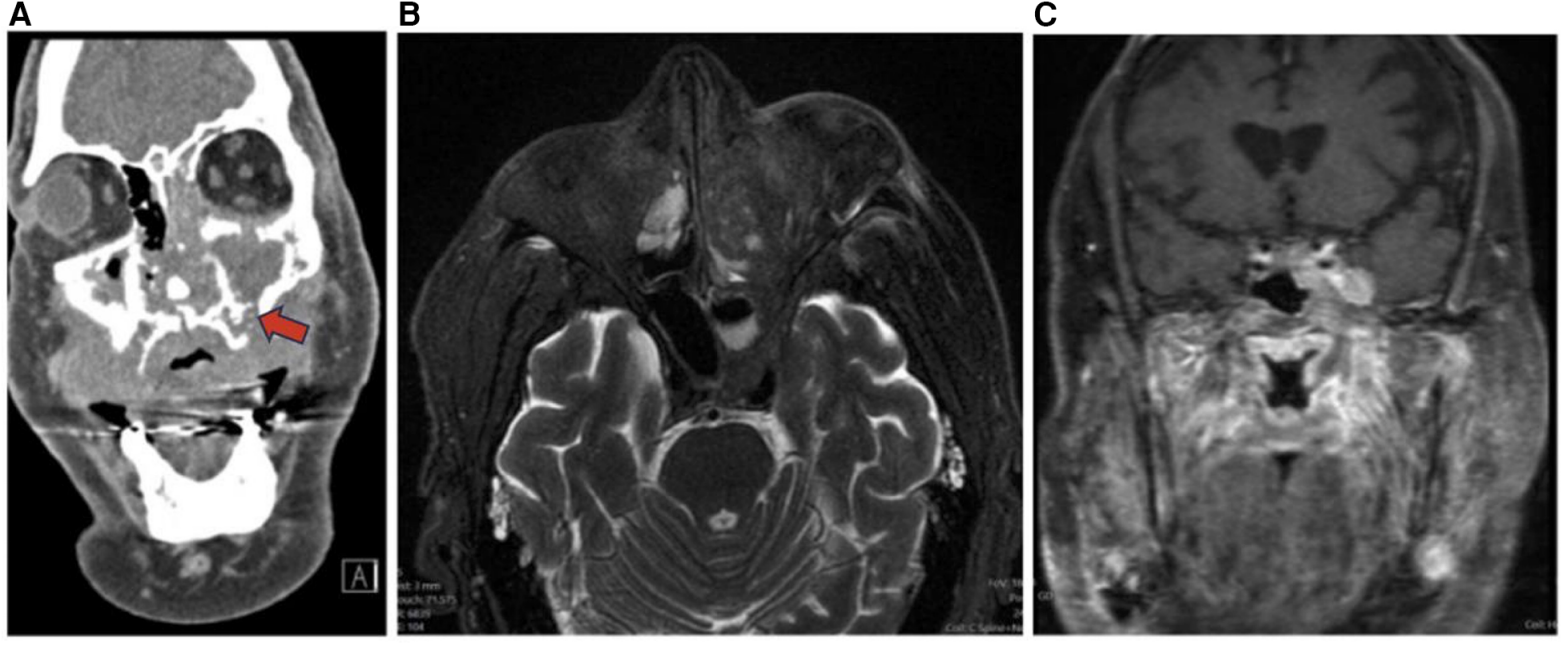
Adenoid cystic carcinoma. Coronal CT **(A)**, axial T2 MRI **(B)**, and coronal T1 + C MRI **(C)** images demonstrating an adenoid cystic carcinoma centered in the left maxillary sinus. The hard palate demonstrates post contrast enhancement and osseous erosion (red arrow).

Adenoid cystic carcinoma (ACC) commonly occurs in women in the 5th–6th decade and accounts for 10% of salivary gland tumors and 5% of sinonasal malignancies. Tubular and cribriform are low-grade morphologies, while solid is a higher-grade tumor (6). ACC is slow growing, relentless, and commonly occurs in the maxillary sinus and nasal cavity with possible infiltration into the sphenoid or ethmoid sinuses (1, 5). ACC is characterized by wide expansile or destructive bone defects and perineural or angioinvasion (Figure 4). The overall recurrence rate of ACC is 56.2% due to local recurrence, lymph node involvement, and late distant metastases to the lungs, liver and bone (1). On MRI, ACC appear as an irregular mass of heterogeneous intensity that may be isointense on T1WI and iso-hyperintense on T2WI with variable amounts of cellularity (1, 6). While MRI is more sensitive for ACC detection, CT can be complementary to MRI for characterizing the extent of bone invasion and perineural spread of head and neck ACC (6, 30).

## Sinonasal undifferentiated carcinoma (SNUC)

**Figure 5:**
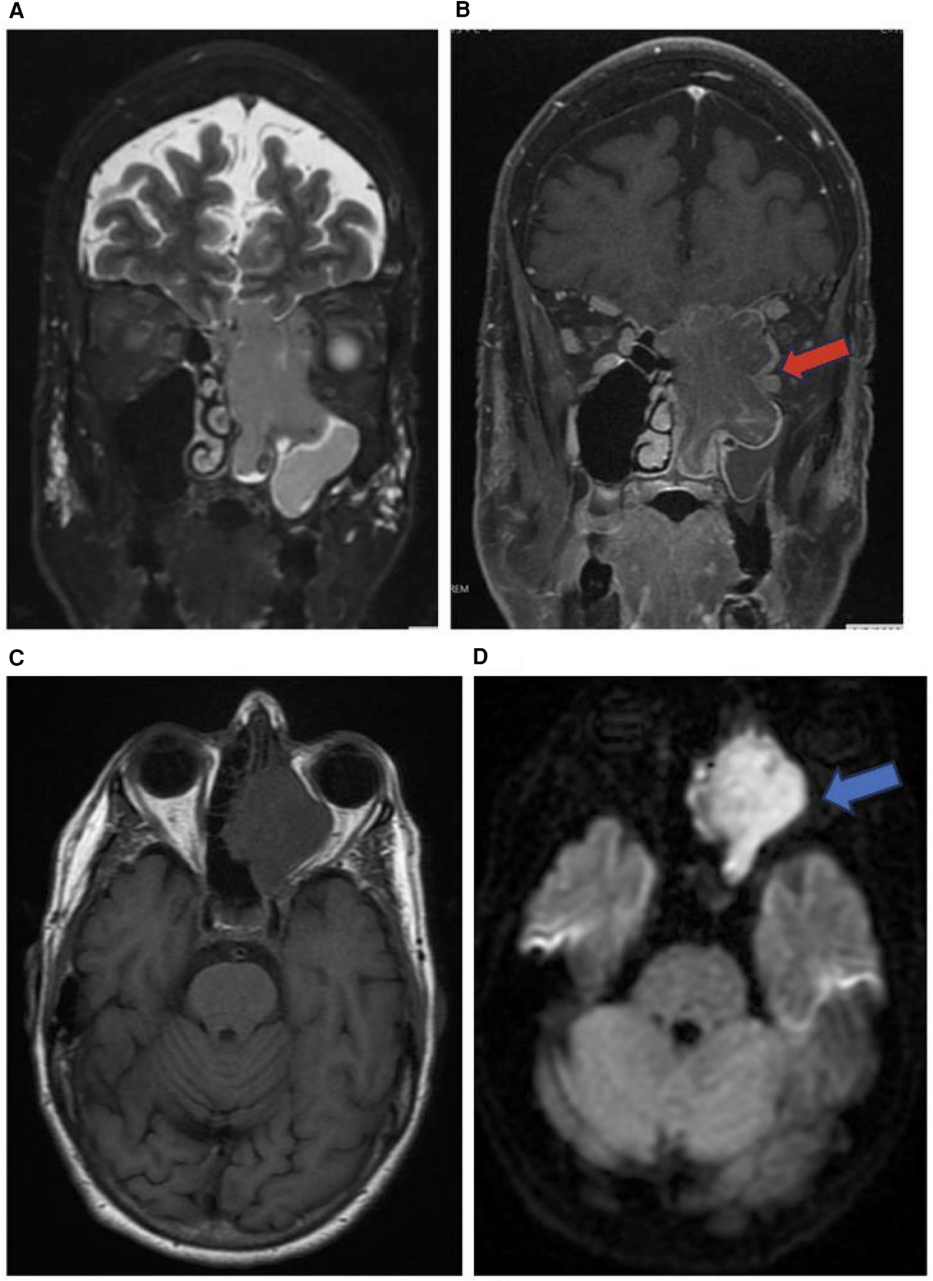
Sinonasal undifferentiated carcinoma. Coronal T2 **(A)**, coronal T1 + C **(B)**, axial T1 **(C)**, & axial DWI **(D)** images showing a mass extending from the left maxillary sinus (red arrow) into the orbit (blue arrow) and intracranial fossa.

Sinonasal undifferentiated carcinoma (SNUC) is a rare tumor with poor overall prognosis which accounts for approximately 3%–5% of sinonasal cancers (7). SNUC is more common in males and often presents as stage IV disease exhibiting aggressive growth, bone destruction, local intracranial and orbital invasion, and distant metastasis (1, 7). SNUC tumors are large (>4 cm) and typically arise in the ethmoid sinus and superior nasal cavity. Local invasion into adjacent structures such as the paranasal sinuses, anterior fossa, orbits, pterygopalatine fossa, parapharyngeal space, and cavernous sinus has been reported (Figure 5) (8). SNUC normally appears as a non-calcified mass with ill-defined margins, bone destruction, variable contrast enhancement, and central necrosis on CT with contrast (8). On T1WI SNUC appears isointense with heterogeneous enhancement on T1 with contrast. On T2WI SNUC appears iso-hyperintense. SNUC and SCC may present with similar non-specific imaging features (1).

## Esthesioneuroblastoma (ENB)

**Figure 6:**
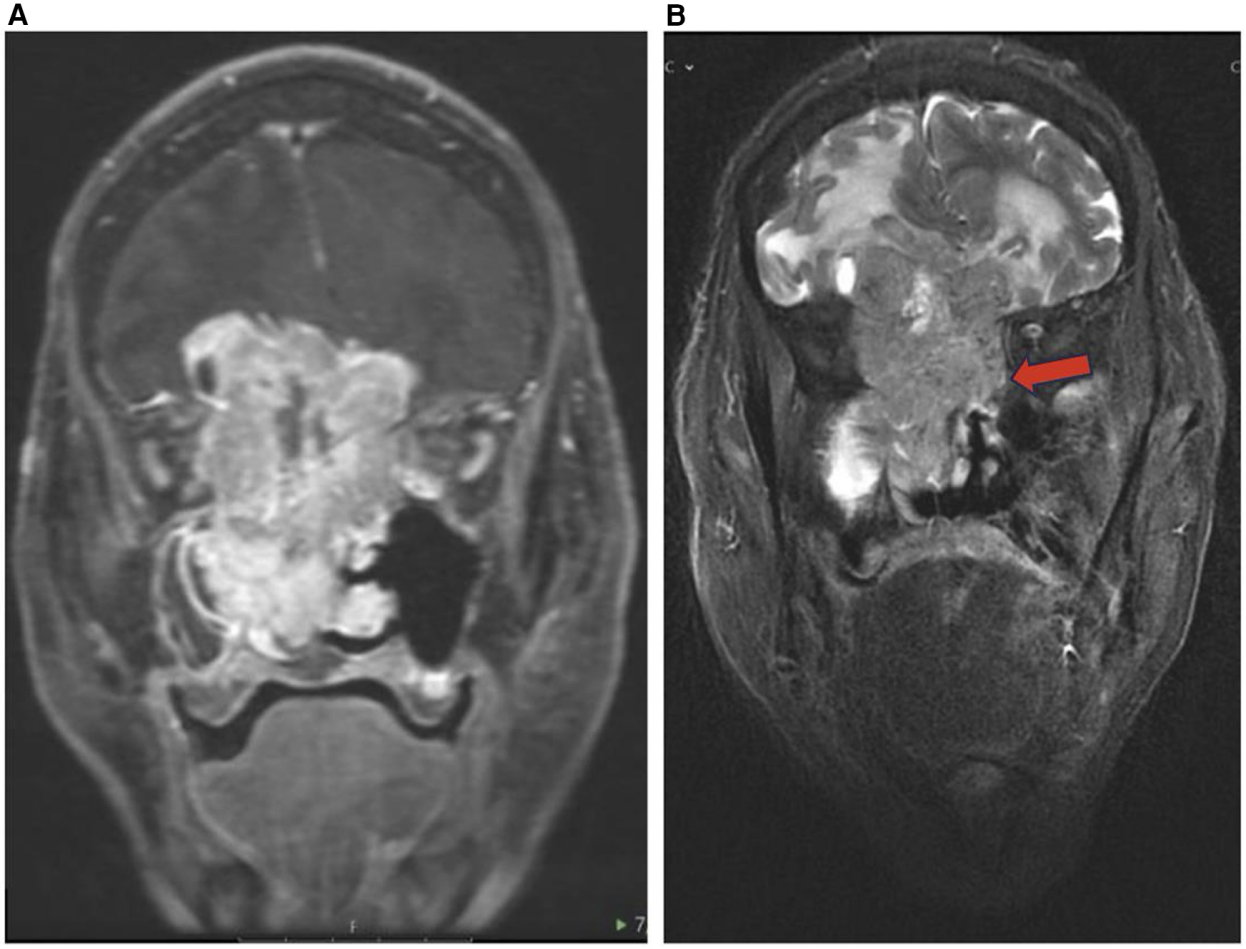
Esthesioneuroblastoma. Coronal T1 + C **(A)** and coronal T2 **(B)** images showing an enhancing cystic lesion in the bilateral fossa representing ENB with extension to adjacent orbits and anterior cranial fossa.

Esthesioneuroblastoma (ENB) is an uncommon dumbbell shaped lesion that accounts for 2%–6% of intranasal cancers and has an incidence rate of 0.4 cases per million population. ENB is a neuroendocrine tumor that arises from the olfactory epithelium in the superior nasal cavity and exhibits aggressive local invasion and metastasis assessed via CT and MRI (Figure 6). ENB can be classified by the conventional tumor, node, metastasis (TNM) system, the Kadish staging system, or the modified TNM Dulguerov staging system. ENB is characterized by slow growth as well as local invasion into the paranasal sinuses, anterior cranial fossa, cribriform plate, and orbit (Figure 6). Metastasis to cervical lymph nodes occurs in >23% of cases and metastasis to the neck, lungs, liver, and bone are also common. CT is used to assess for local invasion and bone involvement in ENB while MRI can better define the subtle extent of local invasion and dural or subarachnoid seeding. ENB normally appears hypointense on T1 weighted MRI and hyperintense on T2 weighted MRI. Incidental findings of cystic intracranial lesions are suggestive of ENB (9).

## Sinonasal melanoma

**Figure 7:**
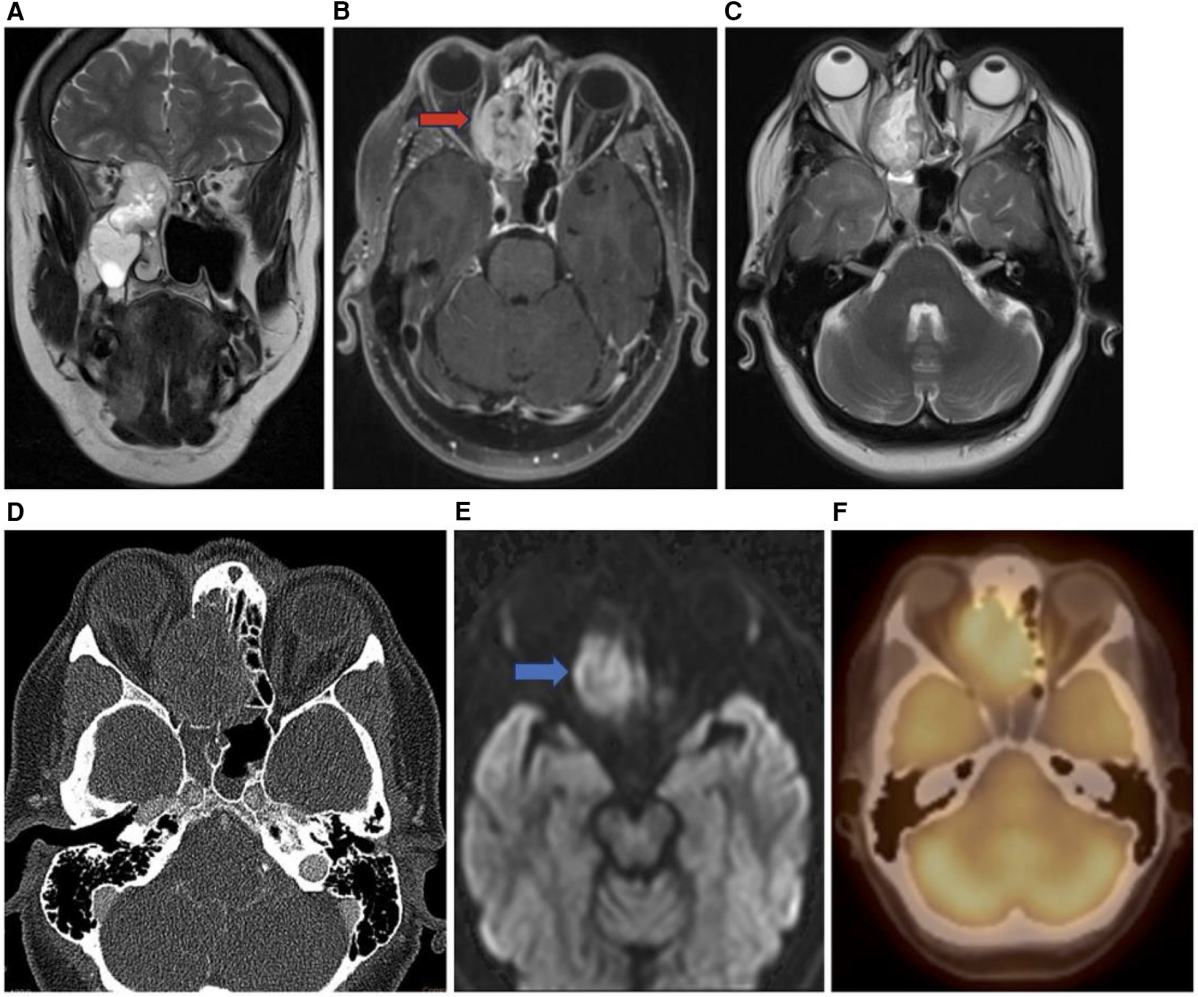
Sinonasal melanoma. Coronal T2 **(A)**, axial T1 + C **(B)**, axial T2 **(C)**, axial CT bone **(D)**, axial DWI **(E)**, and axial PET **(F)** images showing an enhancing sinonasal melanoma centered in the right nasal cavity (red arrow) extending into the adjacent right orbit and into the anterior cranial fossa demonstrating diffusion restriction (blue arrow) and PET activity.

Sinonasal melanoma is a rare malignant lesion that arises from mucosal melanocytes of the nasal cavity and paranasal sinuses and accounts for 0.3% of all melanomas and less than 5% of all head and neck melanomas (10, 11). Sinonasal melanoma has a 5-year survival of <35% and sinus melanoma has a worse prognosis than the more common nasal melanoma (11). The most frequently affected areas are the nasal septum, lateral nasal wall, maxillary antrum, and ethmoid sinuses (Figure 7). In 40%–50% of patients, distant metastases via hematogenous spread to the cervical lymph nodes, liver, skin, lung, bone, or brain are commonly found. Deeply invasive local growth can be seen along with recurrence after treatment (11). Similar to other subtypes of sinonasal tumors CT can be used to define the extent of local invasion and bone involvement of the skull base, orbit walls, and carotid canals (1, 11).

Sinonasal malignant melanoma may present with melanotic or amelanotic patterns. Generally, melanotic patterns present as iso-hyperintense on T1WI and hypointense on T2WI, while amelanotic patterns present as hypo-isointense on T1WI and hyper-isointense on T2WI relative to the cortex (11, 12). Deviations from this pattern are common, and some studies have internal flow voids suggesting vascular flow networks indicative of sinonasal melanoma although this is not a specific diagnostic feature (11).

## Sinonasal lymphoma

**Figure 8:**
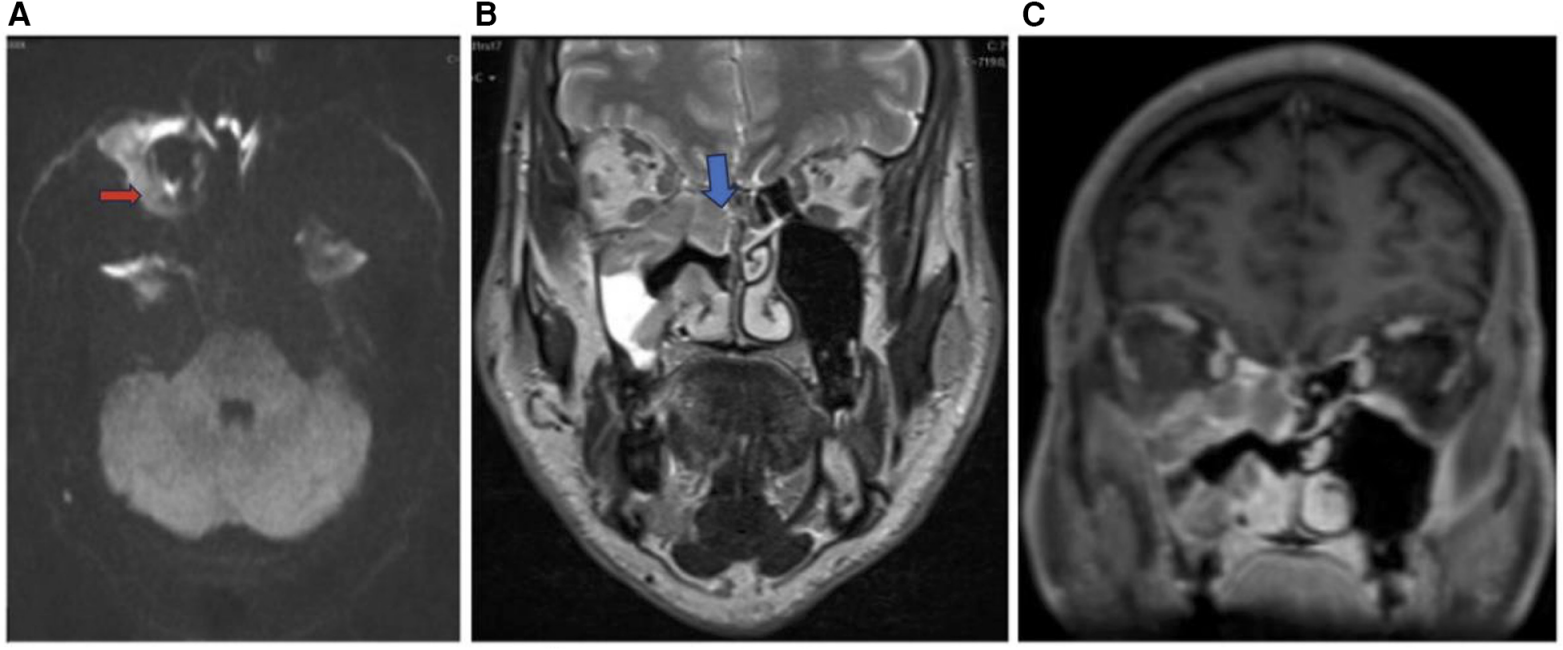
Sinonasal lymphoma. Axial DWI **(A)**, coronal T2 **(B)**, and coronal T1 + C **(C)** images showing sinonasal lymphoma centered in the right maxillary sinus (red arrow) extending to the right ethmoid (blue arrow) and right inferior orbital margin. Pathology confirmed CD5 and CD10 positive germinal center B cell DLBCL.

After SCC, NHL is the most frequently observed malignancy in the sinonasal tract, usually in adults over the age of 60, with recent imaging studies showing regional lymphadenopathy in 7%–19% of cases (1, 19). There are several histological subtypes of sinonasal lymphomas including diffuse large B-cell lymphoma (DLBCL), NK/T cell lymphoma (NKTL), and non-Hodgkin's Lymphoma (NHL). DLBCLs frequently arise from the paranasal sinuses such as the maxillary sinus, with intracranial or orbital involvement (Figure 8). NKTLs most often arise from the nasal cavity with Asian and South American populations disproportionately affected.

Extranodal lymphomas are commonly found in the head and neck region. In particular, sinonasal lymphomas demonstrate infiltrative or permeative bone invasion on CT with variable regional bone destruction (1). MR has shown to be more useful in the diagnosis of sinonasal lymphomas appearing as a mass with soft tissue attenuation (19). These lesions lack specific clinical signs, leading to late diagnosis, and are monitored, rather than surgically treated. On imaging, NKTLs are heterogeneous with intratumoral necrosis and marked enhancement, whereas DLBCL is homogenous with mild enhancement (20). On CT, NHLs that involve permeative bone invasion across the sinus wall demonstrate intra-tumoral sinus wall remnants. NHLs appear isointense on T1 weighted MRI and hyperintense on T2 weighted MRI, and are homogeneously enhanced. NHLs in the maxillary sinus typically have a lower ADC, which is used to differentiate NHLs from SCC (1).

## Sinonasal rhabdomyosarcoma

**Figure 9:**
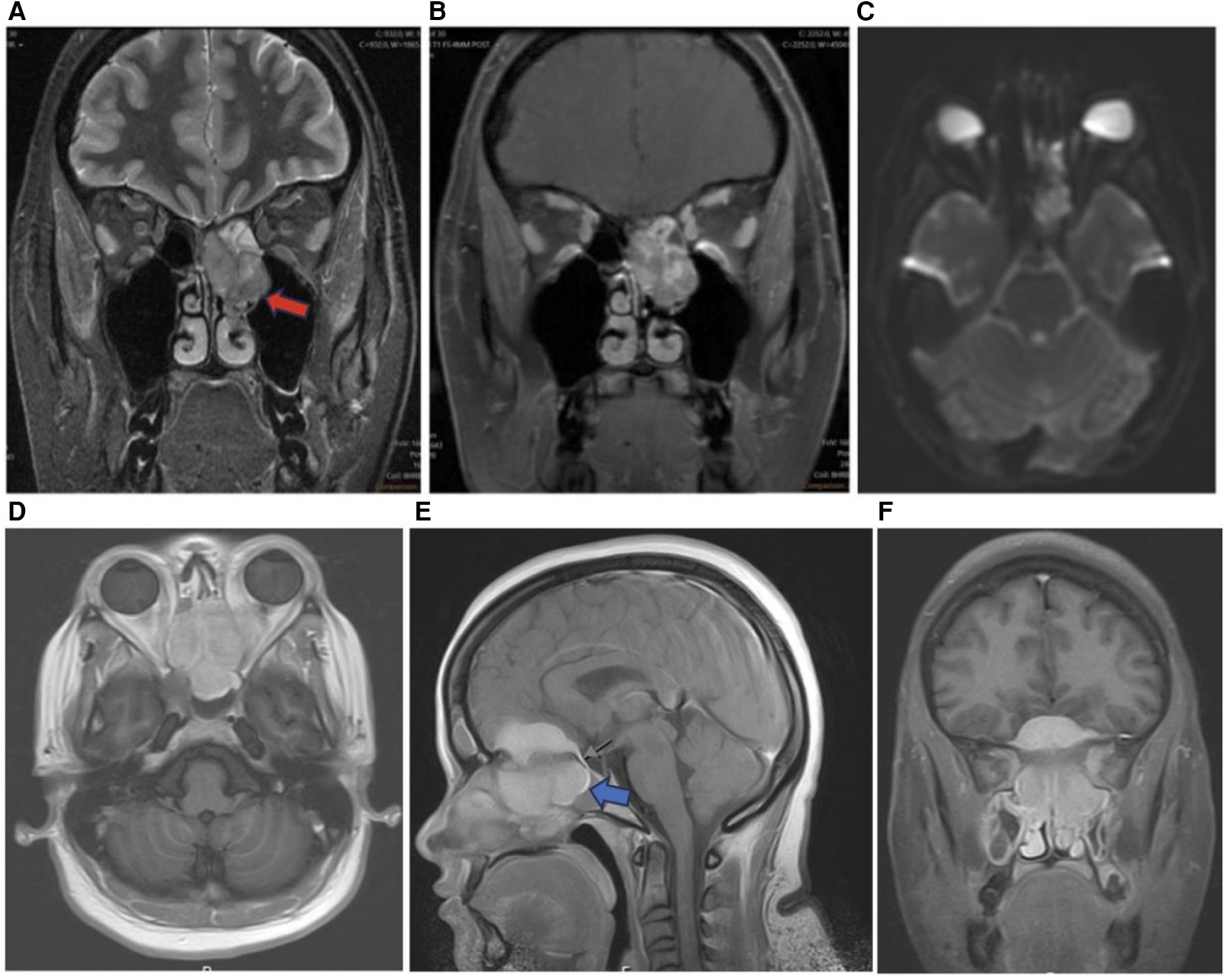
Sinonasal soft tissue tumors. Coronal T2 **(A)**, coronal T1 + C **(B)**, and axial DWI **(C)** showing sinonasal **rhabdomyosarcoma** centered in the left ethmoid (red arrow) demonstrating enhancement and diffusion restriction. Axial T1 + C **(D)**, sagittal T1 + C **(E)**, and coronal T1 + C **(F)** MR images showing a homogenously enhancing **meningioma** centered in the midline of the anterior skull base with extracranial components in the ethmoid sinuses (blue arrow), nasal cavities, sphenoid sinuses, and medial extraconal space of the orbits.

Rhabdomyosarcoma (RMS) is a common soft tissue tumor that constitutes 3%–5% of all childhood tumors. RMS can arise from primitive mesenchymal cells anywhere in the body and 40% of childhood RMS occur in the head and neck region. There are three recognized primary locations for head and neck RMS including parameningeal (50%), non-parameningeal (25%), and orbital (25%). Parameningeal RMS typically have the lowest 5-year overall survival followed by non-parameningeal then orbital locations. The deeper parameningeal location may conceal the tumor until it presents symptomatically at a later stage. Additionally, parameningeal RMS can arise adjacent to vital nearby skull base structures including the internal carotid arteries, cranial nerves, and jugular veins resulting in symptomatic cranial nerve palsy, intracranial extension, and skull base erosion (Figure 9) (21).

RMS can be classified into three main histological subgroups including embryonal, alveolar, and pleomorphic. The embryonal RMS subtype accounts for 70% of cases and typically presents in younger age individuals with a better prognosis. Alveolar RMS accounts for 15% of head and neck RMS and presents in older children with a worse prognosis. The overall clinical presentation depends on the primary tumor location, presence of metastasis, and the histological subtype of the RMS tumor (21).

Imaging is essential for the diagnosis of RMS and assessment of chemotherapy response, recurrence, and distant metastases (21). Head and neck RMS present with bone destruction and extension into adjacent structures. On CT, head and neck RMS present as isodense to hypodense and homogeneously enhancing on CT with contrast. On MRI, head and neck RMS appear isointense to muscle on T1WI and moderately hyperintense to muscle on T2WI (Figure 9). RMS may also demonstrate a botryoid enhancement pattern on MRI (31). On T1-weighted MRI with intravenous contrast, head and neck RMS appear with moderate homogenous enhancement and variable enhancement patterns. Diffusion weighted imaging (DWI) may better allow for identification of RMS in comparison to SCC, as RMS has been shown to have lower mean ADC (32).

## Meningioma

Meningioma are common nonglial CNS tumors accounting for 24%–30% of intracranial tumors arising from arachnoid cap cells (22). Ectopic meningiomas including sinonasal meningiomas arise from tissues that lack meningioma cover and account for <1% of non-epithelial tumors. Primary heterotopic meningioma arises without connection to the cranial nerve foramina, vertebral canal, or intracranial tissue and may present in the sinonasal tract and paranasal sinuses (13). Most ectopic nasal meningioma are typically benign psammomatous type tumors, but may also present as fibrous type tumors. On CT imaging, nasal meningiomas appear uniformly dense with a polyp-like soft-tissue appearance, clear boundaries, smooth edges, and homogenous contrast enhancement (Figure 9) (13). Imaging is crucial to characterize the location, size, and impact of nasal meningiomas on adjacent structures. However, nasal meningioma can be difficult to differentiate using only imaging from other benign tumors in the sinonasal tract such as hemangioma, inverted papilloma, nasal polyps, and fibromas (13). For final diagnosis of ectopic nasal meningioma histopathologic confirmation is needed. Surgical resection including trans-nasal endoscopic resection is the preferred treatment modality for sinonasal meningioma, but the recurrence rate can exceed 25%. Follow-up imaging can be used to monitor for recurrence (13).

## Inverted papilloma

**Figure 10:**
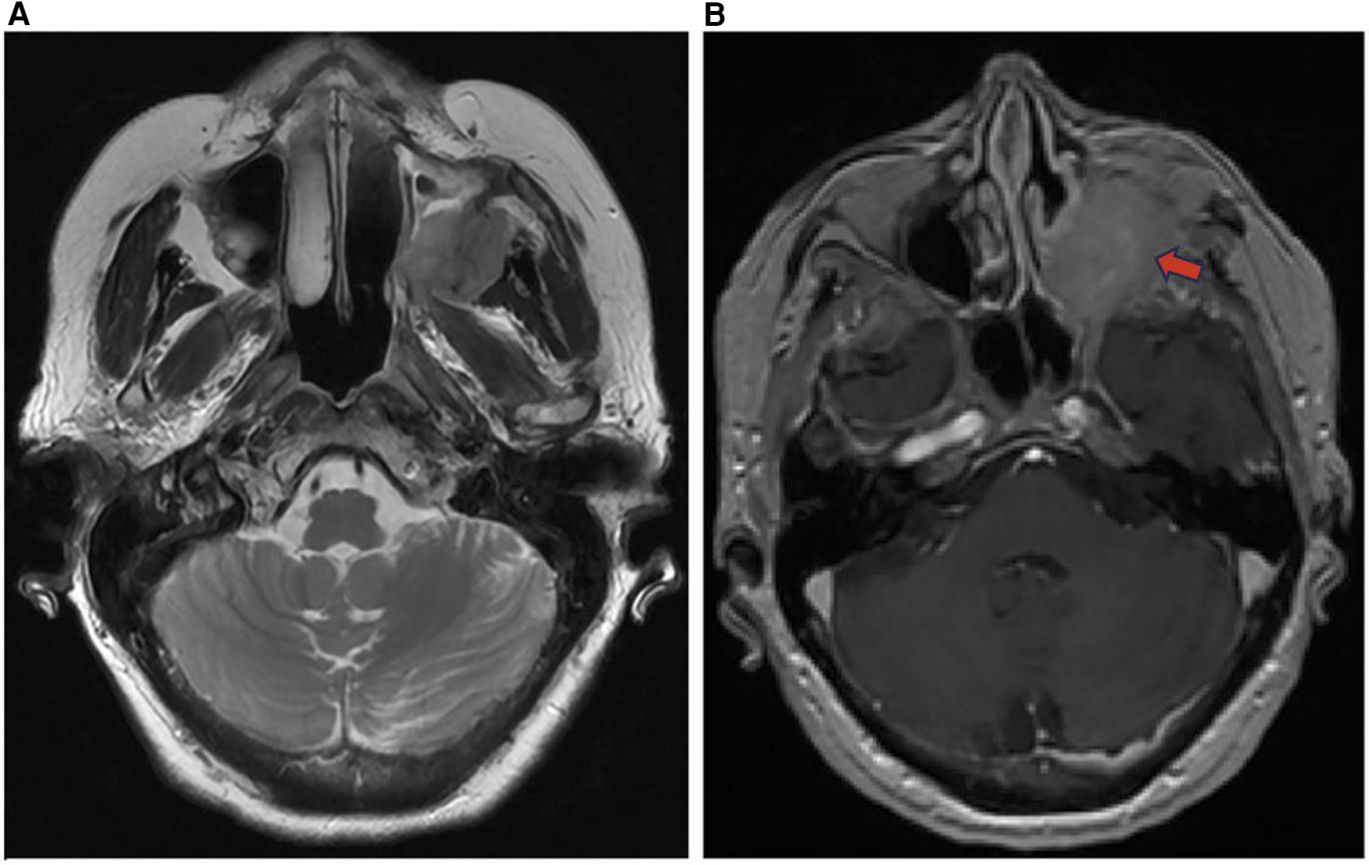
Inverted papilloma axial T2 **(A)** and T1 + C **(B)** images demonstrate an enhancing soft tissue mass of the left maxillary sinus (red arrow) representing an inverted papilloma.

Inverted papilloma (IP) represents 0.4%–4% of sinonasal tumors, originating from the lateral nasal wall near the middle turbinate or ethmoid recesses (14). These are locally aggressive, benign neoplasms, though large IPs can extend into the maxillary sinus, ethmoid sinus, and nasopharynx. IPs often have high recurrence after surgery, with a recurrence rate of 2%–15% (14). Initial evaluation for IPs involves CT for soft tissue masses and bone remodeling rather than bone destruction. MRI + contrast helps differentiate IPs from inflammatory antrochoanal polyps, which display peripheral contrast enhancement. IPs appear iso- to hypointense on T1-weighted MRI and hyperintense to muscle on T2-weighted MRI (Figure 10). A “convoluted cerebriform” pattern on MRI is visualized in up to 80% of IP patients as alternating high and low signals on T2 and T1 weighted MRI with contrast. Though this pattern may also be seen on MRI with malignant neoplasms, it has been shown in retrospective studies to be a reliable MR pattern for IPs (14, 15).

## Antrochoanal polyp

**Figure 11:**
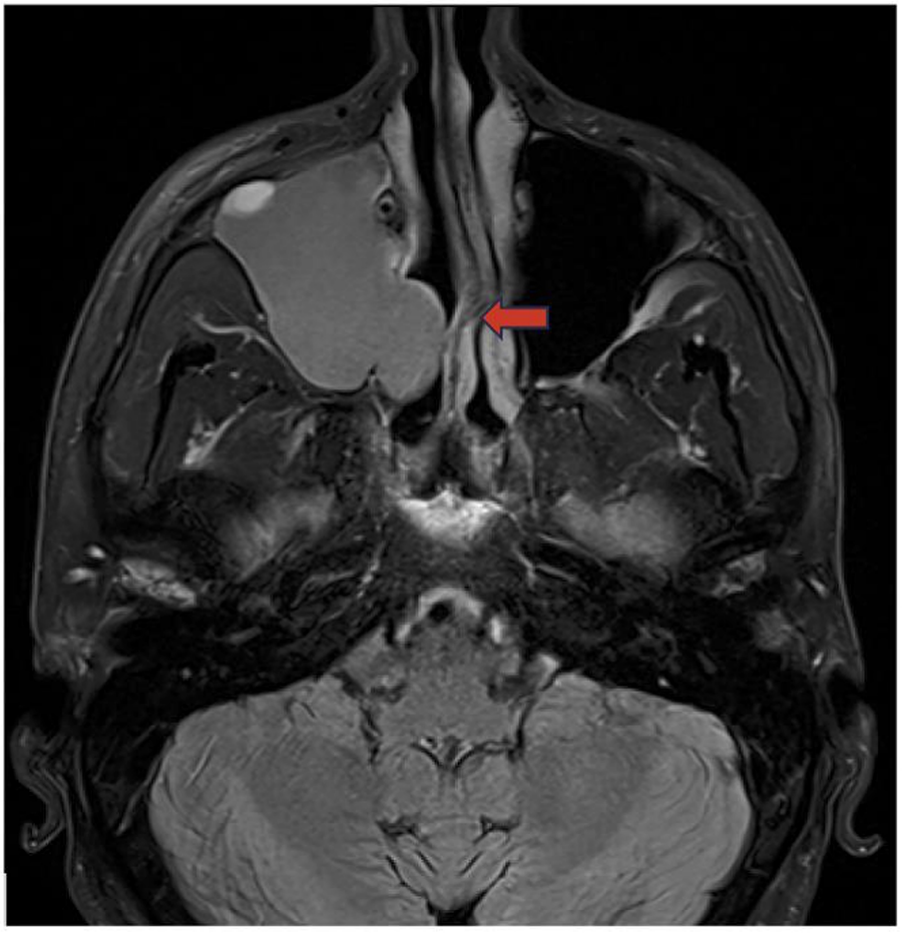
Antrochoanal polyp axial T2 weighted image demonstrates an antrochoanal polyp centered in the right maxillary sinus extending through the osteomeatal unit (red arrow) into the nasal cavity.

Antrochoanal Polyps (AP) are rare, benign mucosal lesions with solid and cystic components. APs typically arise from the maxillary sinus and pass through the ostia to the choana and nasopharynx (Figure 11). These polyps are indicative of chronic inflammation, most often seen in children with allergies, but also in adults with chronic sinusitis (16). Similar to IPs, APs do not involve bone destruction. The only known risk factors are anatomical abnormalities. The most common abnormalities are nasal septal deviation, inferior turbinate hypertrophy, and concha bullosa. Both the solid and cystic polyp components are removed through functional endoscopic sinus surgery (FESS) to reduce complication and recurrence. Despite the high success rate, APs treated with FESS can still have up to a 15% recurrence rate (16, 17, 23). On CT, APs appear as a unilateral hypodense mass from an enlarged opacified maxillary sinus. APs are iso-hyperintense on T1-weighted MRI, iso-hypointense on T2-weighted MRI, and demonstrate peripheral enhancement with contrast administration (16).

## Mucous retention cyst

**Figure 12:**
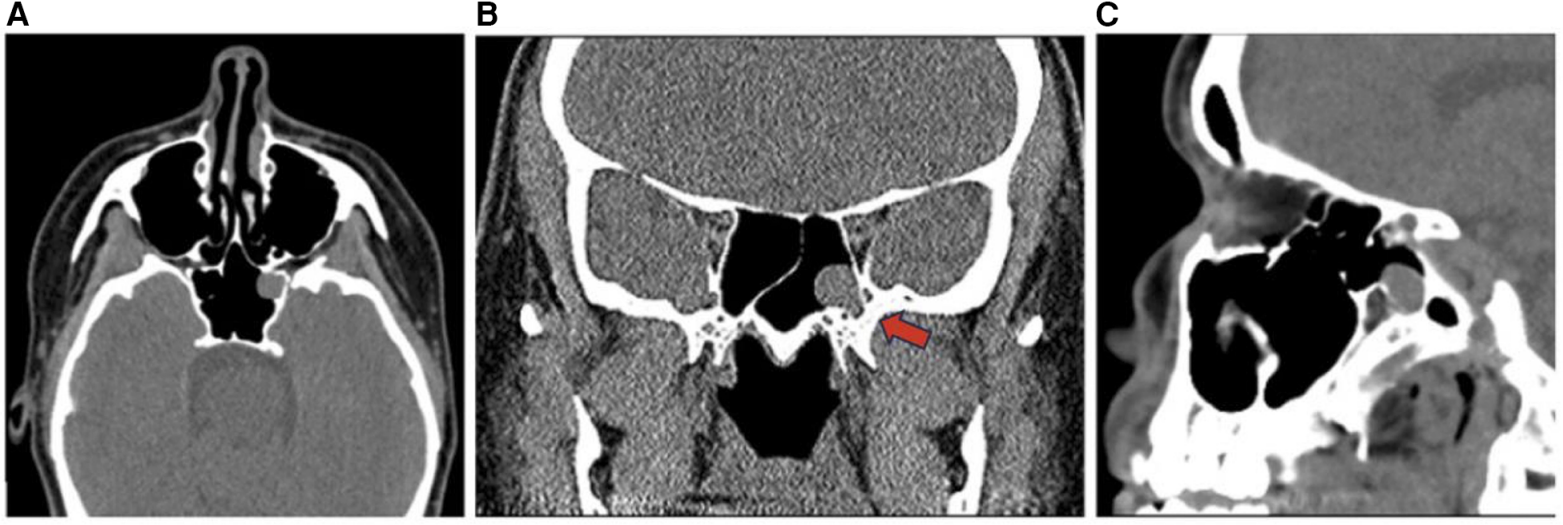
Mucous retention cyst axial **(A)**, sagittal **(B)**, and coronal **(C)** CT head images demonstrate a retention cyst in the left sphenoid sinus (red arrow).

Mucus retention cysts (MRCs) are benign lesions in the paranasal sinuses representing obstructed submucosal mucinous glands. MRCs present similarly to sinonasal polyps, but without extension into the nasal cavity. Though MRCs are often asymptomatic and exhibit spontaneous regression, they can present with headache, periorbital pain, and nasal obstruction, necessitating endoscopic surgical resection. These cysts are reported to occur in 1.4%–9.6% of the general population and are the most common incidental finding in the maxillary sinus (33). In a retrospective analysis of 510 paranasal sinus CT scans, MRCs were found in 29.4% of cases. MRCs are variable to hypointense on T1-weighted MRI and hyperintense on T2-weighted MRI (Figure 12) (33).

## Pleomorphic adenoma

**Figure 13:**
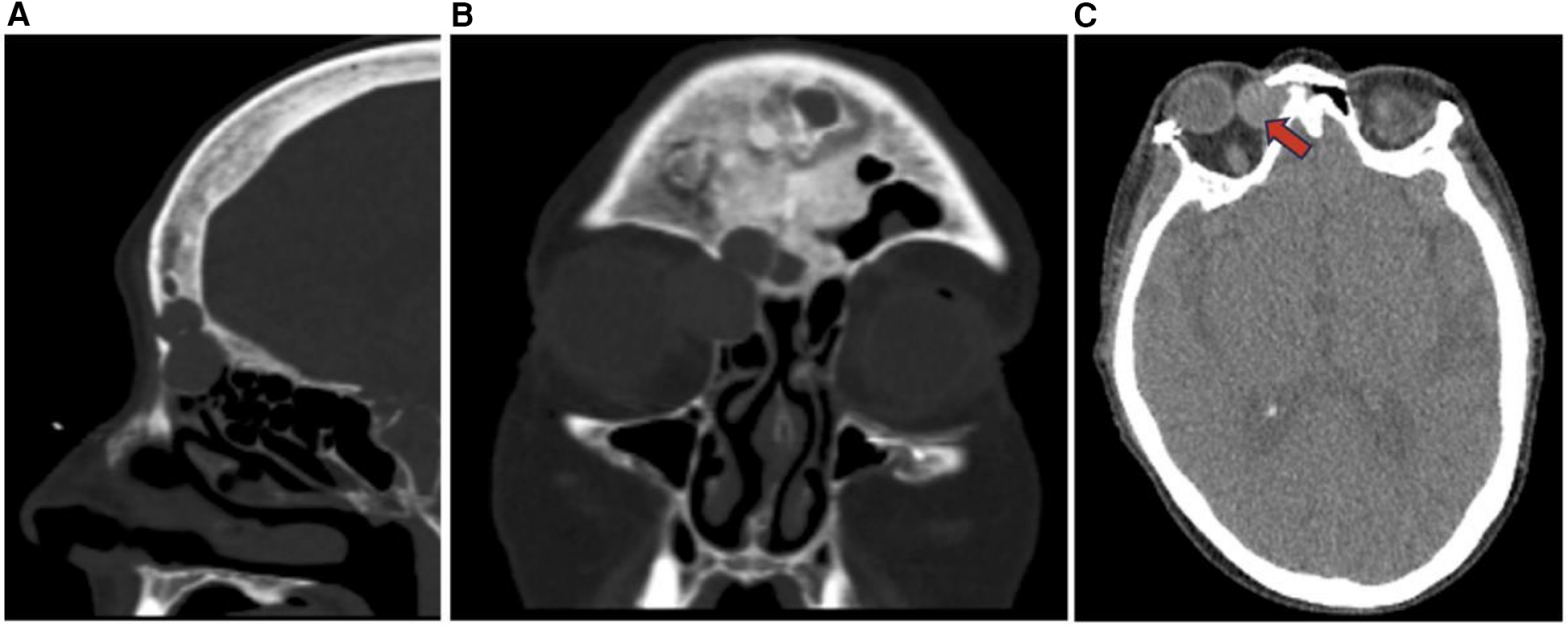
Pleomorphic adenoma sagittal **(A)**, coronal **(B)**, and axial **(C)** CT showing non-destructive expansile soft tissue lesions with cortical thinning in the right paranasal sinuses (red arrow) including the ethmoid sinus and frontal sinus possibly representing a neoplasm such as pleomorphic adenoma.

Pleomorphic adenoma is a benign salivary gland tumor commonly found in the major salivary glands such as the parotid gland and rarely occurs in the sinonasal cavity with an incidence rate of 0.4% (18). Pleomorphic adenoma is more common in females ages 30–60 and is not reported to be associated with occupational or chemical exposures (18). Common presenting symptoms of intranasal pleomorphic adenomas are non-specific including unilateral nasal obstruction, epistaxis, and pain. Intranasal pleomorphic adenomas are often located in the nasal septum, range in size from 0.7 to 7 cm, and may lead to nasal deviation in severe cases. Imaging features include hyperintensity on T2-weighted MRI and bony changes or calcifications on CT (Figure 13). Treatment requires complete surgical excision with negative margins to prevent malignant transformation which may occur in up to 4% of cases and post-surgical recurrence which may occur in up to 8.8% of cases. Post-operative imaging is utilized to monitor for recurrence 1 year after the tumor excision (18).

## Dermoid cyst

**Figure 14:**
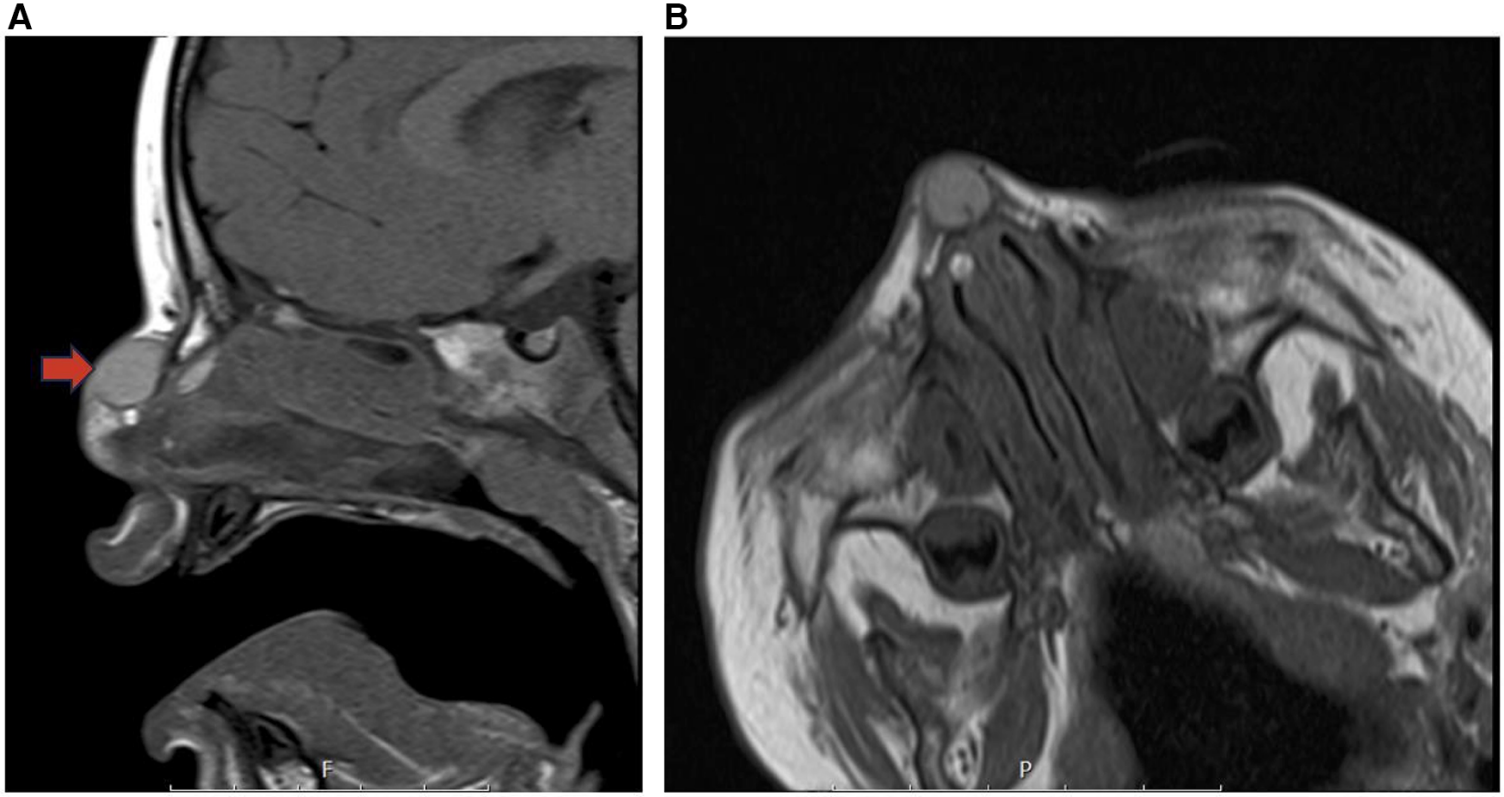
Dermoid cyst sagittal T1 **(A)** and coraxial T1 **(B)** MR images showing a cystic desmoid sinus tract along with a satellite dermoid cyst within the submucosal soft tissues of the right anterior superior nasal septum (red arrow).

Dermoid cysts arise from germ cells that may have undergone aberrant embryonal migration. Nasal dermoid cysts are congenital neuroectodermal tumors that typically present in children prior to the age of 3 and are found in 1–20,000 to 1 in 40,000 individuals accounting for 11%–12% of head and neck dermoid lesions (24, 25). These lesions may exist in the form of a sinus, fistula, or a cyst and in 10%–45% of cases, may extend intracranially potentially leading to meningitis or abscess. Nasal dermoid cysts typically extend along the midline of the nasal bridge from the glabella to the columella and are located in the superficial nasal region in 50% of cases (Figure 14) (25). Dermoid cysts can contain a variety of embryonal tissues including skin, hair, and sweat glands, which distinguishes dermoid cysts from epidermoid lesions. The presence of tracts extending through intra-nasal bone predisposes to infection, but nasal dermoid cysts are typically asymptomatic and found incidentally on imaging, appearing hyperintense on T1-weighted MRI and contrast enhancing with low density on CT (24). CT and MRI are essential to evaluate the extent of the nasal dermoid cyst, whether there is intracranial involvement or abscess formation, and for surgical planning. Treatment consists of surgical resection of the dermoid cyst and associated sinus tracts to reduce the recurrence rate as well as follow up imaging to monitor for recurrence (24).

## Conclusion

General clinical features of sinonasal tumors may include nasal obstruction, headache, epistaxis, diplopia, altered phonation, facial pain, and anosmia, while more specific signs may include palpable masses and neurologic signs. The local spread of sinonasal tumors beyond the sinonasal tract may impact adjacent structures including the internal carotid artery, skull base, and orbit. Diagnostic imaging is essential to determine the extent of local tumor invasion and to identify lymph node involvement as well as distant metastasis to organs such as the lungs, liver, kidney, bone, and brain. Imaging features alongside histological data and the presence of metastatic disease are essential for the staging, prognosis, and treatment planning of sinonasal tumors.

This work seeks to provide a further exploration of a variety of sinonasal tumors as a concise reference for practicing radiologists to review the imaging findings and anatomic impacts of these lesions. The focus of this work is on clearly presenting a variety of sinonasal lesions including malignant and benign tumors with an emphasis on the affected anatomic regions and imaging characteristics associated with each lesion. Table 1 provides a concise overview of the key points presented in this article, and Figure 1 provides a clear medical illustration of the anatomic landmarks in the region of the paranasal sinuses and sinonasal tract, which may be impacted by expanding sinonasal lesions.

Compared to prior work done by Kawaguchi and colleagues, this article seeks to include an additional number of lesions and more up to date information from a variety of published sources including multiple reviews and case reports (1). Other work done be Bracigliano and colleagues, Eggesbo, as well as Reghunath and colleagues also present a variety of sinonasal lesions with a discussion of imaging findings (3, 34, 35). Given our focus on imaging differentiators of sinonasal tumors, other features including the presenting clinical characteristics, histopathologic features, and treatment of each lesion are only briefly discussed and connected to imaging features where applicable.

## References

[1] Kawaguchi M Kato H Tomita H Mizuta K Aoki M Hara A Imaging characteristics of malignant sinonasal tumors. J Clin Med. (2017) 6(12):116. 10.3390/jcm6120116 29211048 PMC5742805

[2] Turner JH Reh DD. Incidence and survival in patients with sinonasal cancer: a historical analysis of population-based data. Head Neck. (2011) 34:877–85. 10.1002/hed.21830 22127982

[3] Bracigliano A Tatangelo F Perri F Di Lorenzo G Tafuto R Ottaiano A Malignant sinonasal tumors: update on histological and clinical management. Curr Oncol. (2021) 28(4):2420–38. 10.3390/curroncol28040222 34287240 PMC8293118

[4] Razek AA Sieza S Maha B. Assessment of nasal and paranasal sinus masses by diffusion-weighted MR imaging. J Neuroradiol. (2009) 36(4):206–11. 10.1016/j.neurad.2009.06.001 19577300

[5] Leivo I. Sinonasal adenocarcinoma: update on classification. Immunophenotype and Molecular Features. Head Neck Pathol. (2016) 10:68–74. 10.1007/s12105-016-0694-9 26830399 PMC4746143

[6] Sepúlveda I Platin E Delgado C Rojas P. Sinonasal adenoid cystic carcinoma with intracranial invasion and perineural spread: a case report and review of the literature. J Clin Imaging Sci. (2015) 5:57. 10.4103/2156-7514.168710 26664774 PMC4647119

[7] Xu CC Dziegielewski PT McGaw WT Seikaly H. Sinonasal undifferentiated carcinoma (SNUC): the Alberta experience and literature review. J Otolaryngol Head Neck Surg. (2013) 42(1):2. 10.1186/1916-0216-42-2 23663264 PMC3646548

[8] Phillips CD Futterer SF Lipper MH Levine PA. Sinonasal undifferentiated carcinoma: CT and MR imaging of an uncommon neoplasm of the nasal cavity. Radiology. (1997) 202(2):477–80. 10.1148/radiology.202.2.9015077 9015077

[9] Dublin AB Bobinski M. Imaging characteristics of olfactory neuroblastoma (esthesioneuroblastoma). J Neurol Surg B Skull Base. (2016) 77(1):1–5. 10.1055/s-0035-1564053 26949582 PMC4777621

[10] Gore MR Zanation AM. Survival in sinonasal melanoma: a meta-analysis. J Neurol Surg B Skull Base. (2012) 73(3):157–62. 10.1055/s-0032-1301400 23730543 PMC3424013

[11] Wong VK Lubner MG Menias CO Mellnick VM Kennedy TA Bhalla S Clinical and imaging features of noncutaneous melanoma. AJR Am J Roentgenol. (2017) 208(5):942–59. 10.2214/AJR.16.16800 28301211

[12] Escott EJ. A variety of appearances of malignant melanoma in the head: a review. Radiographics. (2001) 21(3):625–39. 10.1148/radiographics.21.3.g01ma19625 11353111

[13] Hu X Jiang M Feng Z Wang J Wang P Cai J. Primary heterotopic meningioma of nasal cavity: case report and literature review. Ear Nose Throat J. (2022) 101(9):383–8. 10.1177/0145561320974863 33215534

[14] Chawla A Shenoy J Chokkappan K Chung R. Imaging features of sinonasal inverted papilloma: a pictorial review. Curr Probl Diagn Radiol. (2016) 45(5):347–53. 10.1067/j.cpradiol.2015.10.004 26632214

[15] Eid M Eissa L. Imaging of sino-nasal inverted papilloma: how can we emphasize the usefulness of the “striated pattern” sign? Egypt J Radiol Nucl Med. (2020) 51(29):1–16. 10.1186/s43055-020-0134-4

[16] Chagarlamudi K O’Brien WT Towbin RB Towbin AJ. Antrochoanal polyp. Appl Radiol. (2019) 48(1):38–40. 10.37549/AR2554

[17] Pagella F Emanuelli E Pusateri A Borsetto D Cazzador D Marangoni R Clinical features and management of antrochoanal polyps in children: cues from a clinical series of 58 patients. Int J Pediatr Otorhinolaryngol. (2018) 114:87–91. 10.1016/j.ijporl.2018.08.033 30262373

[18] Mead KM Kuhar H Ibrahim N Adelman M Zehr B Schoenfield L Pleomorphic adenoma of the nasal cavity: a case study. Otolaryngol Case Rep. (2022) 23(100408):2468–5488. 10.1016/j.xocr.2022.100408

[19] Bitner BF Htun NN Wang BY Brem EA Kuan EC. Sinonasal lymphoma: a primer for otolaryngologists. Laryngoscope Investig Otolaryngol. (2022) 7(6):1712–24. 10.1002/lio2.941 36544932 PMC9764779

[20] Chen Y Wang X Li L Li W Xian J. Correction to: differential diagnosis of sinonasal extranodal NK/T cell lymphoma and diffuse large B cell lymphoma on MRI. Neuroradiology. (2020) 62(9):1201. 10.1007/s00234-020-02488-8 32617604 PMC7645479

[21] Freling NJ Merks JH Saeed P Balm AJM Bras J Pieters BR Imaging findings in craniofacial childhood rhabdomyosarcoma. Pediatr Radiol. (2010) 40(11):1723–38. 10.1007/s00247-010-1787-3 20725831 PMC2950273

[22] Abir M Rihab L Bellakhdhar M Malika O Jihen H Wassim K A rare case of primary sinonasal meningioma: a case report. Int J Surg Case Rep. (2022) 99:107620. 10.1016/j.ijscr.2022.107620 36122423 PMC9568705

[23] Lee DH Yoon TM Lee JK Lim SC. Difference of antrochoanal polyp between children and adults. Int J Pediatr Otorhinolaryngol. (2016) 84:143–6. 10.1016/j.ijporl.2016.03.004 27063770

[24] Kadasah S Alhelali A Aldhabaan S Qahtani AA Musleh A Alshahrani A Nasal dermoid cyst with Sinus tract intranasal bone: a case report. Int J Otolaryngol Head Neck Surg. (2024) 13(2):149–56. 10.4236/ijohns.2024.132014

[25] Lee DH Yoon TM Lee JK Lim SC. Dermoid cyst of nasal septum in an adult patient: a case report. Medicine (Baltimore). (2018) 97(45):e13028. 10.1097/MD.0000000000013028 30407296 PMC6250529

[26] Sanghvi S Khan MN Patel NR Yeldandi S Baredes S Eloy JA. Epidemiology of sinonasal squamous cell carcinoma: a comprehensive analysis of 4994 patients. Laryngoscope. (2014) 124(1):76–83. 10.1002/lary.24264 23775607

[27] Elgart K Faden DL. Sinonasal squamous cell carcinoma: etiology, pathogenesis, and the role of human papilloma virus. Curr Otorhinolaryngol Rep. (2020) 8(2):111–9. 10.1007/s40136-020-00279-6 32582473 PMC7314379

[28] Chowdhury N Alvi S Kimura K Tawfik O Manna P Beahm D Outcomes of HPV-related nasal squamous cell carcinoma. Laryngoscope. (2017) 127:1600–3. 10.1002/lary.26477 28271500

[29] Doescher J Piontek G Wirth M Bettstetter M Schlegel J Haller B Epstein-Barr virus infection is strictly associated with the metastatic spread of sinonasal squamous-cell carcinomas. Oral Oncol. (2015 Oct) 51(10):929–34. 10.1016/j.oraloncology.2015.07.008 26272275

[30] Uraizee I Cipriani NA Ginat DT. Adenoid cystic carcinoma of the oral cavity: radiology-pathology correlation. Head Neck Pathol. (2018) 12(4):562–6. 10.1007/s12105-017-0849-3 28879643 PMC6232209

[31] Liu JH Qi M Huang WH Sha Y Zhang F. The magnetic resonance characteristics of sinonasal rhabdomyosarcoma in adults: analysis of 27 cases and comparison with pathological subtypes. BMC Med Imaging. (2023) 23(1):98. 10.1186/s12880-023-01062-x 37507673 PMC10375770

[32] Wang X Song L Chong V Wang Y Li J Xian J. Multiparametric MRI findings of sinonasal rhabdomyosarcoma in adults with comparison to carcinoma. J Magn Reson Imaging. (2017) 45(4):998–1004. 10.1002/jmri.25484 27648498

[33] Bal M Berkiten G Uyanık E. Mucous retention cysts of the paranasal sinuses. Hippokratia. (2014) 18(4):379. PMCID: . PMID: . 26052215 PMC4453822

[34] Reghunath A Mittal MK Thukral BB Sinha M. Approach to sinonasal masses: a comprehensive review. J Head Neck Physicians Surg. (2022) 10(1):14–25. 10.4103/jhnps.jhnps_10_22

[35] Eggesbø HB. Imaging of sinonasal tumours. Cancer Imaging. (2012) 12:136–52. 10.1102/1470-7330.2012.0015 22571851 PMC3362868

